# The amygdala–insula–medial prefrontal cortex–lateral prefrontal cortex pathway and its disorders

**DOI:** 10.3389/fnana.2022.1028546

**Published:** 2022-11-24

**Authors:** Dorit Ben Shalom

**Affiliations:** School of Brain Sciences and Cognition, Ben-Gurion University of the Negev, Beersheba, Israel

**Keywords:** amygdala, insula, mPFC, LPFC, emotion

## Abstract

Smith and Lane have suggested a model of emotion processing with at least three stations: areas like the amygdala, which process discrete body features areas like the anterior insula, which process whole-body patterns and areas like the medial prefrontal cortex, which process emotion concepts. Ben Shalom and Bonneh have suggested a model of the prefrontal cortex, in which medial BA 9 integrates emotional states, and lateral BA 9 performs selection/inhibition on these states. Taken together, the current paper suggests a pathway for emotion processing with at least four stations: areas like the amygdala, which process discrete body features areas like the anterior insula, which process whole-body patterns, medial BA 9 which integrates emotion concepts, and lateral BA 9, which performs selection/inhibition on these concepts. Following the existing literature, it then suggest that there is a significant involvement of the amygdala in psychopathy (Blair), of the anterior insula in alexithymia (Bird), of the medial BA 9 in deficits in somatosensory discrimination (Ben Shalom), and of lateral BA 9 in emotional impulsivity (Ronel).

## Introduction

**FIGURE 1:**
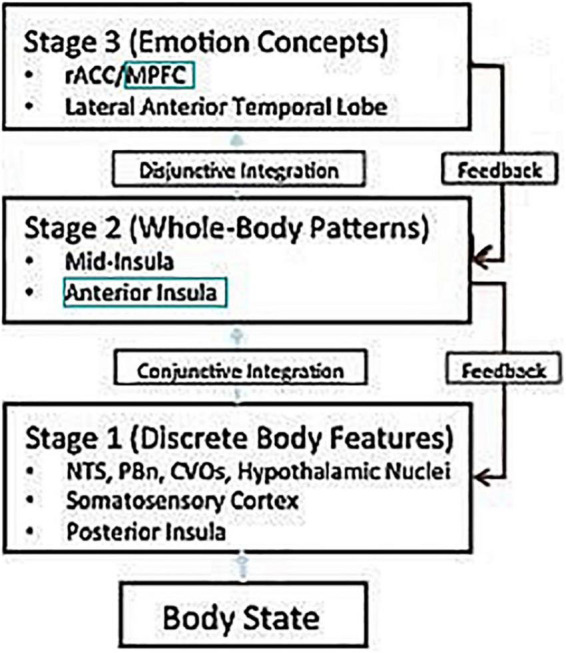
Adapted from Smith and Lane (2015). NTS, nucleus of the solitary tract; PBn, parabrachial nucleus; CVOs, circumventricular organs; rACC, rostral ACC; MPFC, medial prefrontal cortex.

The current paper can be seen as either an extension of Smith and Lane (2015) model of emotional processing, or as an application of Ben Shalom and Bonneh (2019) model of the prefrontal cortex. Either way, one ends up with a pathway of four stations: the amygdala, insula, medial prefrontal cortex, and lateral prefrontal cortex. Smith and Lane (2015) model of emotion processing talks about three types of emotion representations: Stage 1 (discrete body features), such as in the posterior insula, and presumably the amygdala; Stage 2 (whole body patterns), such as the anterior insula; and Stage 3 (emotion concepts), such as in the medial prefrontal cortex. In other words, it proposes a pathway with at least three consecutive stations: the amygdala, the anterior insula, and the medial prefrontal cortex (Figure 1).

**FIGURE 2:**
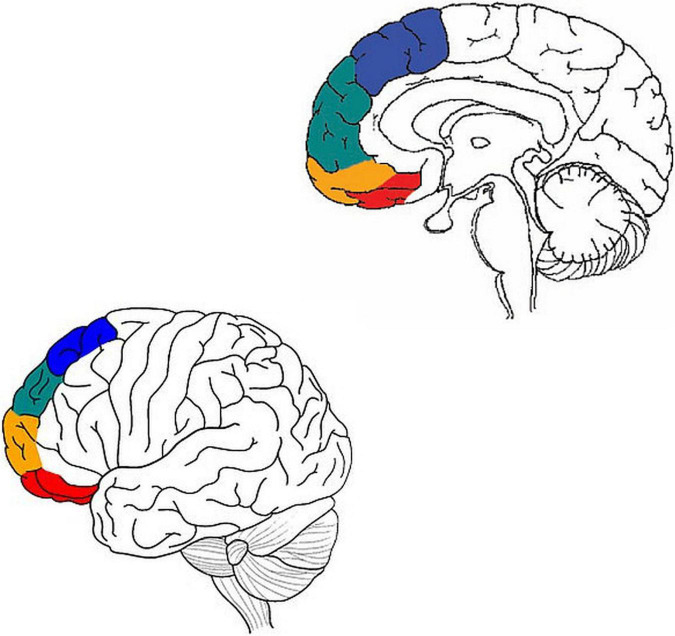
Adapted from Ben Shalom (2009) and Ronel (2018). Motor processing in blue, emotion in green, memory in orange, and sensory in red.

Ben Shalom and Bonneh (2019) suggest a model of the narrow prefrontal cortex (BA 8, 9, 10, 11) in terms of two divisions: horizontal and vertical. But while their horizontal division is traditional (medial vs. lateral), their vertical division is new: four streams of information, from dorsal to ventral (motor, emotion, memory, and sensory). Within each stream, the medial prefrontal cortex integrates basic cognitive objects, while the lateral prefrontal cortex performs selection/inhibition on these objects. In other words, it proposes a pathway with at least two consecutive stations: the medial prefrontal cortex, and the lateral prefrontal cortex (Figure 2).

Putting these two models together, one gets a pathway with at least four consecutive stations: Stage 1 (discrete body features), such as in the posterior insula, and presumably the amygdala; Stage 2 (whole body patterns), such as in the anterior insula; Stage 3 (integration of emotion concepts) such as medial BA 9; and Stage 4 (selection/inhibition of emotion concepts), in lateral BA 9.

## The amygdala and psychopathy

Psychopathy is a personality disorder characterized by an emotional dysfunction (reduced guilt and empathy) whose antecedents can be identified in a subgroup of young people showing severe antisocial behavior (Hare, 2003). Even though we now know that it correlates with dysfunction in several brain regions (De Brito et al., 2021), it is still accepted that a major defining feature of the disorder is dysfunction of the amygdala (Blair, 2008; Marsh et al., 2013): the amygdala is involved in the formation of both positive and negative stimulus associations. Individuals with psychopathy show impairment in stimulus reinforcement learning (whether positive or negative), which is crucial for learning that some social things are bad to do. As such, these individuals are more likely to learn to use antisocial strategies to achieve their goals. In addition, the reduced amygdala responsivity leads to reduced empathy. Finally, the impairment in positive stimulus learning may relate to the reduced attachment reported in this disorder (Hare, 2003); individuals with psychopathy may find their carers to be less positive stimuli and thus be less motivated to seek their company.

## The anterior insula and alexithymia

Alexithymia has been described as a subclinical phenomenon marked by difficulties in identifying and describing feelings and difficulties in distinguishing feelings from the bodily sensations of emotion (Bird et al., 2010). The argument for connecting alexithymia to dysfunction of the anterior insula comes from both functional and structural sources (Smith et al., 2020). In terms of _function_, alexithymia is associated with reduced anterior insula activation on several emotional tasks, such as when rating the emotional valence of stimuli from the International Affective Pictures System (Silani et al., 2008), or when observing either emotional facial expressions (Kano et al., 2003; Reker et al., 2010) or the sight of others in pain (Bird et al., 2010; Feldmanhall et al., 2013). In terms of _structure_, alexithymia is associated with reduced anterior insula volume (Borsci et al., 2009; Ihme et al., 2013; Bernhardt et al., 2014), and reduced coherence of the structural connections of the anterior insula. A recent study (Hogeveen et al., 2016) found _acquired_ alexithymia following damage to the anterior insula.

## Medial BA 9 and deficits in somatosensory discrimination

In contrast, there is considerable evidence that the medial prefrontal cortex is involved in the processing of basic conscious feelings. For example, Phan et al. (2002) reviewed 55 PET and fMRI studies of the processing of basic conscious feelings (happiness, fear, anger, sadness, and disgust), and concluded the following: that while every basic feeling has its own associated areas, the one area that was in common to all of them was the medial prefrontal cortex (BA 9/10). Thus, a problem with medical BA 9 would lead to impaired emotion concepts, and a difficulty in reading the anterior insula body maps, even if the body maps themselves are in fact intact.

But the deficit is probably even more general. For example, somatosensory discrimination relates to the discrimination capacities of the tactile and proprioceptive modalities, derived from somatosensory information regarding touch, pressure, vibration, temperature, texture, pain, and the location and movement of body parts (Bröring et al., 2008).

A recent scoping review (Zetler et al., 2019) found that most studies of people with ASD (a disorder proposed to involve the medial prefrontal cortex, Ben Shalom, 2009; Uddin, 2011) showed atypical somatosensory discrimination, especially among young children. In other words, a difficulty in discriminating basic feelings can be a special case of a difficulty in discriminating body states, whether they are emotional or not.

## Lateral BA 9 and emotional impulsivity

Finally, there is much evidence supporting a relation between lateral BA 9 and emotional impulsivity, or, more generally, emotion regulation. One piece of evidence comes from studies of addiction, which is often assumes to be related to emotional impulsivity. For example, a study by Chen and Mo (2017) compared regional homogeneity in nicotine addicts and control participants. The nicotine addicts had lower regional homogeneity values in a prefrontal area whose peak coordinates were in lateral BA 9. Similarly, a post-mortem analysis of individuals with alcohol use disorder demonstrated that DNA methylation alterations in the lateral BA 9 are associated with (and might result in) increased risk of alcohol use disorders (Wang et al., 2016). Another piece of evidence comes from the study of emotion regulation strategies such as reappraisal and suppression (Ronel, 2018): Compared to passive viewing conditions, both reappraisal (Xiong et al., 2013; Hallam et al., 2014; Rabinak et al., 2014), as well as suppression (Hallam et al., 2014), were found to show greater brain activation in lateral BA 9. In addition, two different meta-analyses have been used to examine fMRI studies of emotion regulation. Buhle et al. (2014) found that reappraisal consistently activated lateral BA 9; Frank et al. (2014) found that such reappraisal was accompanied by increased activation in lateral BA 9 together with reduced activation in the amygdala.

## Data availability statement

The original contributions presented in this study are included in the article/supplementary material, further inquiries can be directed to the corresponding author.

## Author contributions

DB wrote the manuscript, contributed to the article, and approved the submitted version.
